# Skin-conformable printed supercapacitors and their performance in wear

**DOI:** 10.1038/s41598-020-72244-8

**Published:** 2020-09-16

**Authors:** Anna Railanmaa, Ayat Soltani, Suvi Lehtimäki, Nazanin Pournoori, Jari Keskinen, Mikko Hokka, Donald Lupo

**Affiliations:** 1 Faculty of Information Technology and Communication Sciences, Tampere University, Tampere, Finland; 2 Faculty of Engineering and Natural Sciences, Tampere University, Tampere, Finland

**Keywords:** Energy storage, Supercapacitors, Electronic devices

## Abstract

Wearable sensors and electronic systems are of great interest these days, but their viability depends on the availability of compatible energy storage solutions. Such sensors can either be integrated into clothing or attached directly to the skin, each case presenting a different set of requirements for the devices. In this work, we examine the performance of printed supercapacitors while attached to the skin. The devices are manufactured from benign materials, such as water, carbon and sodium chloride, and worn on the forearm or chest for 24 h for durability testing. The supercapacitors exhibit excellent mechanical durability and stay well attached under all test conditions. Electrically, the supercapacitors exhibit reliable capacitive function throughout the test period; other key parameters such as equivalent series resistance and leakage current are affected but to a minimal extent. The movement and deformation of the supercapacitor show good compatibility with the skin, as shown by the Digital Image Correlation full field strain measurements on and around the capacitor. The supercapacitors deform with the skin and do not hinder normal movement or function.

## Introduction

Wearable electronics has been a topic of much interest in recent years, as has its subdiscipline epidermal electronics, where the electronic devices are worn directly on the skin. Especially in medical device research, there has been interest in patch-type devices^1–7^, which eliminate the need for rigid packaging and impractical hanging wires from patient monitoring systems. The literature in the field is focused on sensing and measuring, but for independent function in the future, these patches will require a compatible energy source as well. It is essential that all components in the measurement system are skin compatible and conformable, including the energy storage components. Supercapacitors^8^ are a good candidate for powering these types of devices, since the active materials can be biocompatible in skin contact. They can be printed onto flexible substrates and easily integrated with other printed components^9–14^. Several efforts to produce wearable and stretchable supercapacitors have been reported^15–18^, but so far the reports concerning the durability of the device when worn on the skin are very limited^19,20^, and the existing reports present very short term trials^21,22^. With these types of devices, the required wear times can range from minutes to hours to even a full day of continuous monitoring. Thus, wearable energy storage devices should be studied over sufficient time periods under realistic end-use conditions before they can be implemented into larger systems.

High stretchability of these wearable electronic devices is frequently emphasized in publications, but in many real-life applications, the general flexibility and malleability of the devices can be more important than tremendous stretchability, especially if design considerations for e.g. device placement are taken account of. In devices integrated into clothing or textiles^23–25^ it can be crucial for the device to stretch with the garment it is attached to. However, in many epidermal applications great stretchability may not be an extremely high priority compared to the general comfort of wear and proper device design.

We report here skin conformable supercapacitors characterized for their actual capability to withstand everyday wear and changes in their performance caused by extended test periods in real-life conditions. The novel device structure combines non-toxic materials with malleable packaging and skin-friendly attachment, which makes them an ideal candidate for epidermal use cases with no health hazards. The samples were subjected to severe stressors during the test periods and their effect on the performance of the samples was evaluated by electrical and visual characterization. The results were compared to the performance of unstressed reference samples. We also report a quantitative assessment of patient comfort of the devices when worn on the skin. The assessment was carried out using Digital Image Correlation (DIC) to measure full field deformations of the skin with and without the device attached. The full field strains and the overall mechanical response of the skin with the supercapacitor were compared with the response of the skin as is, and with commercially available products, such as surgical tapes, which offer clinically demonstrated patient comfort.

## Experimental

### Sample preparation

The supercapacitors were printed onto thermoplastic polyurethane (TPU, Platilon) substrates. First, the TPU was laminated at 130 °C for 40 s onto a more rigid PET carrier film in order to improve the structural integrity of the prints while drying the ink. A double layer of TPU was used to improve the water barrier of the substrate in order to retain the aqueous electrolyte within the device for longer in the ambient environment. The current collectors were then printed onto the substrate with Henkel 407C graphite ink by stencil printing with a 120 µm stainless steel stencil. The prints were cured at 100 °C for four hours to ensure that no solvent remained in the structure. After drying, the carrier film was removed and half of the prints were laminated with Opsite Flexifix, which is a thin TPU film with a skin-friendly adhesive on one side. Activated carbon (AC) ink was printed onto one end of the current collector with a 60 µm stainless steel stencil. The AC ink was prepared in house by first dissolving 1.7 g of chitosan (from shrimp shell, low viscosity, Sigma-Aldrich) and 0.7 g of acetic acid into 70 ml of deionized water. Once the chitosan was completely dissolved, 30.9 g of AC powder (Kuraray YP-80F) and an additional 20 ml of water were thoroughly mixed in. The AC prints were allowed to dry in ambient conditions for minimum of 24 h. All prints were cut to size and weighed before and after applying the AC ink in order to determine the specific capacitance from the total electrode mass.

**Figure 1:**
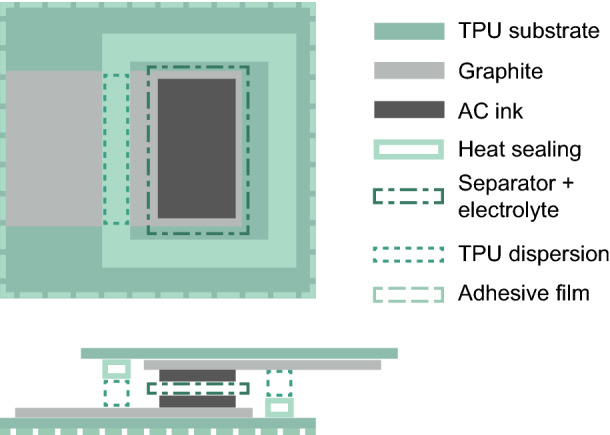
Schematic drawing of the supercapacitor structure. Two identical electrodes are laminated in a face-to-face configuration with the current collectors exposed at each end. The current collector is 30 by 20 mm, the electrode is 10 by 18 mm and the thickness of the entire device is approximately 0.8 mm. The layer thicknesses are not to scale.

For the assembly, a bead of Edolan TPU dispersion was applied on a hot plate at 70 °C onto the midsection of the graphite current collector to enable heat sealing the devices all the way around the electrodes. After this, the electrodes were wetted with an excess of 1 M (aq) sodium chloride electrolyte and a double layer of Dreamweaver Silver AR40 separator was laid on top of one of the electrodes. This was done to prevent potential short circuits caused by a local puncture through the separator. Since the equivalent series resistance (ESR) depends mostly on the current collector^26^, the additional separator layer does not have a significant adverse effect on the electrical properties. The devices were then heat sealed in a head-on configuration with an impulse sealer on all four sides around the electrode and the seams were further secured with a soldering iron right next to the current collector edges. The ends of the current collectors were left exposed for measurement contacts. A schematic drawing of the entire structure is presented in Fig. 1; the process flow has been graphically detailed in the Supplementary Information. The formfactor of the devices is similar to previously reported works^13,26,27^, allowing direct comparison between different materials, manufacturing techniques and levels of deformability. SEM images of the current collector and electrode structure have previously been reported by Keskinen et al.^28^.

### Durability testing and electrical characterization

For the durability testing, the samples were attached to the forearm or the upper chest and worn in everyday use for 24 h at a time. The experiments were approved by the Ethics Committee of the Tampere Region. During the 24 h period, the samples were subject to normal movement of the skin and in many cases also various levels of external pressure while e.g. carrying items or during sleep. The base level of the electrical properties was measured for all samples before attaching the samples to the skin. After being worn for 24 h, the samples were measured again with the same program. A maximum of three samples could be worn and measured simultaneously. Thus, the testing was conducted in multiple batches and every batch included also reference samples, which were not attached to the skin but manufactured at identical conditions and measured at identical intervals compared to the samples attached to the skin and worn by a person. In total, 14 samples were tested in wear along with their reference counterparts.

Because of the soft nature of the substrate, the samples could not be removed from the skin before the final measurement without significant risk of structural damage to the current collectors or electrodes. Thus, the final measurement rounds for determining the effects of wearing the device were conducted while the samples were still attached to the skin. All connections were carefully insulated with surgical tape (Transpore, 3M) from the person wearing the samples in order to avoid any electrical contact between the person and the electronics.

**Figure 2:**
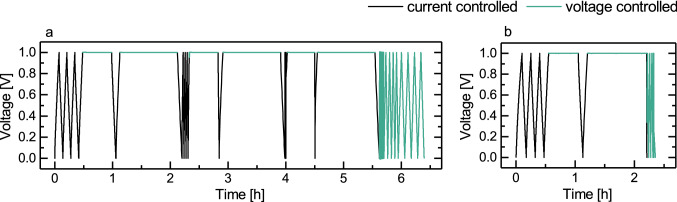
The longer (**a**) and shorter (**b**) measurement protocols. In the protocol a there are three charge/discharge cycles followed by 30 min and 60 min voltage holds. These elements are repeated at three different currents: 1, 3 and 10 mA. The protocol b is identical with 1 mA charge/discharge and voltage holds until the discharge after 1 h hold, which occurs at 10 mA. Data is from a true measurement.

The supercapacitors were electrically characterized with a Maccor 4300 test station (Maccor Inc., USA). Two measurement protocols of different durations were used during the characterization: initial study of the functionality and performance was conducted with a longer version in accordance with a supercapacitor measurement standard^29^ to ensure that the samples were comparable to earlier studies, and a shorter version was modified from this protocol for the measurements of the supercapacitors worn on the skin. The capacitance was determined from the slope of the voltage decrease over time when discharging at a constant current, and the ESR from the initial voltage drop at the beginning of the constant-current discharge. Schematics of the protocols are presented in Fig. 2.

In the shorter protocol, the capacitance was measured after the first 30 min hold period during a 1 mA discharge section. The leakage current was measured from the end of the 1 h hold, and the ESR was measured immediately after that, as the supercapacitor was discharged with 10 mA. Correspondingly, in the longer protocol capacitance was measured after the first 1 h hold at 1 mA discharge. The ESR and leakage current were measured from the 10 mA section; the ESR was extracted from the discharge after 30 min hold and leakage current from the end of the 1 h hold.

In addition, the electrical characterization protocol was performed on the epidermal supercapacitors inside a Vötsch VT4021 climate chamber, in order to obtain a reference point for the effect of the body temperature on the capacitance. The samples were first measured in room temperature and then again at 32 °C.

### Digital image correlation

**Figure 3:**
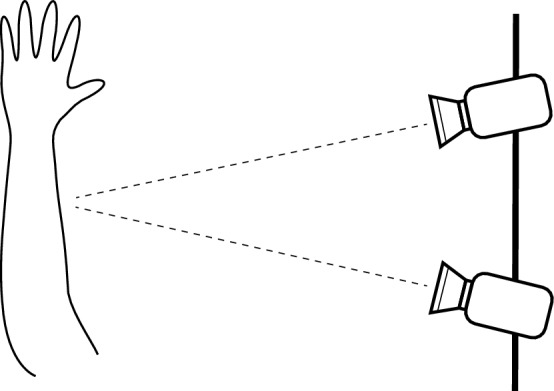
Schematic of the DIC measurement. The subject was perpendicular to the camera axis during imaging.

The effect of the supercapacitor on the movement and stretching of the skin was measured, visualized and quantified using a Digital Image Correlation system^30^, which is a contact free optical method suitable for studying the full field surface displacements and strains on 3D objects. DIC has been previously used to study deformation of various human tissues, e.g. heart, eyes and tendons^31–34^, as well as the skin^35,36^. The supercapacitors were attached to the forearm as in the electrical characterization study and painted first uniformly black, after which a white random speckle pattern was created with a sponge. DIC tracks the movement of the speckle pattern with two cameras, forming a 3D image of the object. Thus, the full field in-plane strains and 3D-displacements can be observed in space over time. The movement of the skin and the supercapacitor were imaged with two 5 mega pixel E-lite cameras and the data were analyzed with the Davis software by Lavision; the system was calibrated with a known 3D calibration target. A schematic picture of the DIC setup is shown in Fig. 3. The cameras were placed approx. 1 m away from the subject, perpendicular to the arm. In addition to the supercapacitor, some commercially available skin tapes were used as a reference for acceptable minimum mobility: a thicker LDPE surgical tape (Transpore, 3M), a thin TPU sheet (Opsite Flexifix, Smith & Nephew) and a perforated non-woven fabric tape (Mefix, Mölnlycke Health Care Oy). A rotation of the wrist was chosen to be the observed motion, as this appeared to produce the most deformation. The experiments were approved by the Ethics Committee of the Tampere Region.

## Results

The samples were not visibly affected by the durability testing. Situations placing the most stress on the devices were pressure from carrying items and walking with hands touching the sides of the body, as well as abrasion from changing clothes. Additionally, compression during sleep, especially in case of the chest samples, was observed. The adhesive held the sample to the skin uniformly around all edges and no peeling off occurred at any point. The devices were not found to hinder normal movements and were practically unnoticeable when in an upright neutral position. In the case of the chest samples, this could potentially be further improved by placing the device even higher on the chest, so that it is not visible to the end user^37^. Removal of the samples caused minor redness only momentarily, and no complications on the skin were observed.

While aqueous sodium chloride is an ideal choice of electrolyte in terms of end-user safety in an application such as this, the devices were monitored during testing in case of liquid electrolyte leaking out of the devices, but no leakage was observed. However, it should be noted that in this type of device, even with modern materials, the barrier properties and the user comfort (wearability) of the device are in direct conflict with each other. A thicker substrate would provide better barrier properties against both oxygen entering the device and the solvent escaping. Simultaneously, increased substrate thickness would make the device bulkier, less deformable and in general reduce the comfort of wear. Hence, the thickness of the substrate is a compromise between comfortability, shelf life and, in this case, printability. The TPU substrate used in this study resulted in the devices degrading due to electrolyte loss in 4–5 days, demonstrating the importance of not using potentially hazardous organic solvent-based electrolytes. There is rarely a need to monitor a patient continuously for more than a day at a time, and in storage the devices can easily be held in sterilizable aluminum coated pouches, which will increase the shelf life significantly.

**Figure 4:**
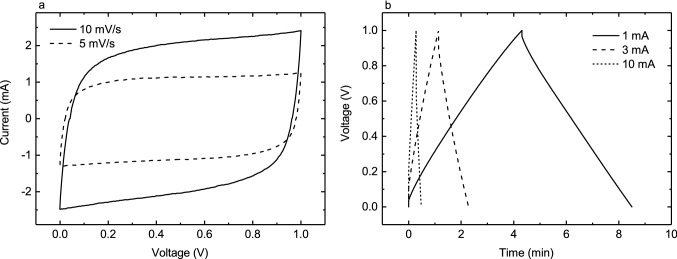
CV (**a**) and charge–discharge (**b**) curves for an average epidermal supercapacitor at various measurement rates.

Cyclic voltammetry (CV) data offers a qualitative assessment of the supercapacitor properties: samples exhibit good capacitive function with moderate ESR. Pseudocapacitive behaviors, including leakage current, are limited and especially at slower charging rates the charge–discharge curves are very linear. CV and charge–discharge plots for an average sample are presented in Fig. 4. More detailed measurement data were extracted from the charge–discharge measurements described. Summarized results for capacitance, leakage current and ESR are presented in Table 1.

**Table 1:** Average performance of the epidermal supercapacitors after wearing them on skin for 24 h with the average change from the measurement before wear.

	After 24 h		Change from original	
	On skin	Reference	On skin	Reference
C [F/g]	26.65	26.89	+ 0.69	+ 0.19
Leakage current [µA]	13.91	15.44	+ 1.32	+ 1.46
ESR [Ω]	22.53	23.22	− 0.16	− 0.14

Corresponding values for the reference samples are also given.

**Figure 5:**
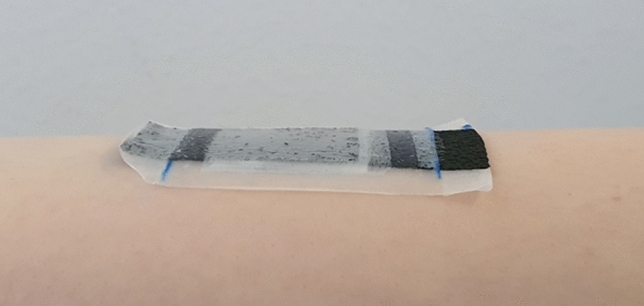
Sideview of an attached sample, illustrating the exposed connection sites.

Leaving the ends of the current collectors exposed to the environment is necessary for performing the measurement without introducing external contact resistances to the characterization of a single component. However, this also means that the ends are not fixed to a substrate or the skin as they would have to be once integrated into an actual application, leaving the current collectors more susceptible to external damage than would be likely in actual use cases. Additionally, the alligator clips of the measurement device form an interface of soft and hard material where they are attached to the current collector, likely producing additional structural damage to the print during any movement. Due to this, the measurement set-up can be considered as a worst-case scenario for the current collectors, as they become subject to extreme bending and folding of the print, which would be highly unlikely to occur in an integrated device. The attachment is illustrated in Fig. 5. The effects of the mechanical stress should manifest particularly in a steep increase of the ESR if the current collector is cracked or otherwise damaged, especially since the current collector has been estimated to form the majority of the ESR of the entire device^26^.

Despite the harsh conditions faced by the connection sites, the ESR of the skin-mounted supercapacitors does not deteriorate significantly in use. In fact, on average the ESR of the worn samples and the references changes quite similarly (− 0.16 Ω and − 0.14 Ω, respectively). In the reference samples, the results are more uniform, whereas the worn samples exhibit a wider distribution of the relative changes in ESR, but this takes place equally in both directions. However, considering the originally relatively large variation of absolute ESR values (17–26 Ω) between the hand-printed samples, the changes induced by the durability testing are negligible. In general, the samples fare well in the durability testing in terms of the resistance, and no significant deterioration can be assigned to the stresses induced by wearing the devices.

The leakage currents range from approx. 10 to 14 µA before wear and 10–20 µA after wear (10–19 µA and 13–19 µA for references, respectively). In general, the references show similar behavior as the worn samples, without major differences. The distribution of the changes in leakage current for the worn samples in the after-wear measurement is slightly wider, but simultaneously the average increase is smaller than for the references. In general, the results are within such a small range that no unambiguous effect of the wearing of the device can be determined from these experiments. It appears that the increase and unreliability in the leakage current is mostly caused by the sealing method and the substrate, rather than damage caused by the wearing of the device.

The leakage current results based on the measurement method enforced by the wearing conditions demonstrate how important the measurement protocol is for the achieved results. The longer 8-h protocol has been utilized in earlier studies^13,26,38^. However, because samples cannot be removed from the skin before the final measurement, a shorter version was required. This change manifests as a systematically higher leakage current (2.8 µA on average) than with the longer version, as the burn-in period is significantly shorter. Thus, in any future reporting the protocol should be covered in sufficient detail in order to allow for comparisons. Capacitance and ESR results were not notably affected by the change in the duration of the protocol.

The capacitance of all the specimens in the study was found to increase slightly as the devices age during the measurement protocol, as seen also in previous studies^13,26^. The specific capacitance of the samples ranges from 21 to 30 F/g (component capacitance divided by the total electrode mass) and at the most the capacitance improves by approximately 2 F/g; in no samples does the capacitance decrease. In the specimens worn on skin, the capacitance increases slightly more than in the reference samples: on average, the specific capacitance of the reference specimens increases by 0.19 F/g, whereas in the specimens worn on the skin the average increase was 0.69 F/g. This was hypothesized to partially arise from the temperature differences between the skin mounted and reference specimens, since the samples attached to the body are a few degrees warmer than the room temperature reference specimens: 29 °C on the arm and 32 °C on the chest. A temperature-controlled reference measurement was performed in order to study the effect of the temperature difference on this capacitance increase. The samples measured in a climate chamber at 32 °C exhibited a systematic increase of approximately 0.35 F/g in capacitance from room temperature to the body temperature. This indicates that some of the capacitance increase does indeed result from the elevated temperature when mounted on skin; however, it does not explain all of it.

In general, the arm-mounted devices all experience a somewhat higher increase in capacitance than the chest mounted samples, even though the samples on the chest were slightly warmer. The few anomalies, where capacitance increases significantly more than the average, are also arm samples. A likely cause for this is external pressure applied onto the device, which could cause the electrolyte to penetrate the pores of the AC particles better than in samples which are not under similar pressure.

**Figure 6:**
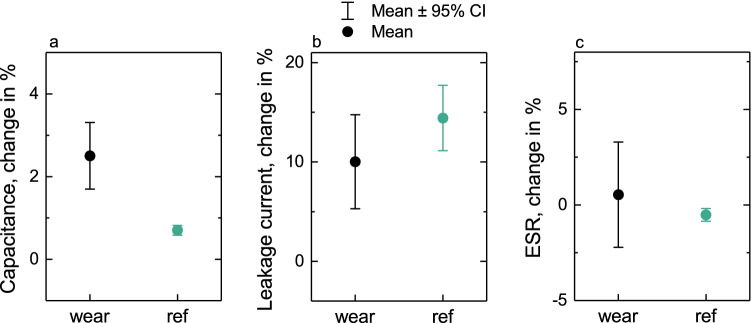
Percentual changes over the wear test period in capacitance (**a**), leakage current (**b**) and ESR (**c**) for worn samples and the references.

Percentual changes observed in capacitance, leakage current and ESR have been summarized in Fig. 6. Considering the electrical characterization results, the skin-compatible printed supercapacitors perform equally to more robustly packaged similar devices both before and after wear^11,13,26^. Leakage current of the wearable devices is slightly higher due to the relatively poor barrier quality of the TPU, but it still does not compromise the function of the supercapacitor. Capacitance and ESR are within a similar range as reported with other printed AC supercapacitors^11,13,26^, and modifications to enable wearability have not compromised the good electrical performance. Longer term voltage holds or charge–discharge cycles may lead to further degradation of the active materials in a supercapacitor^39^. On skin measurements of longer voltage hold periods are challenging to execute, but the previous investigations of fundamental properties of the same active electrode materials have demonstrated that the printed supercaparitor can be expected to withstand considerably longer hold times and charge–discharge cycles^11,26^ than demonstrated here.

**Figure 7:**
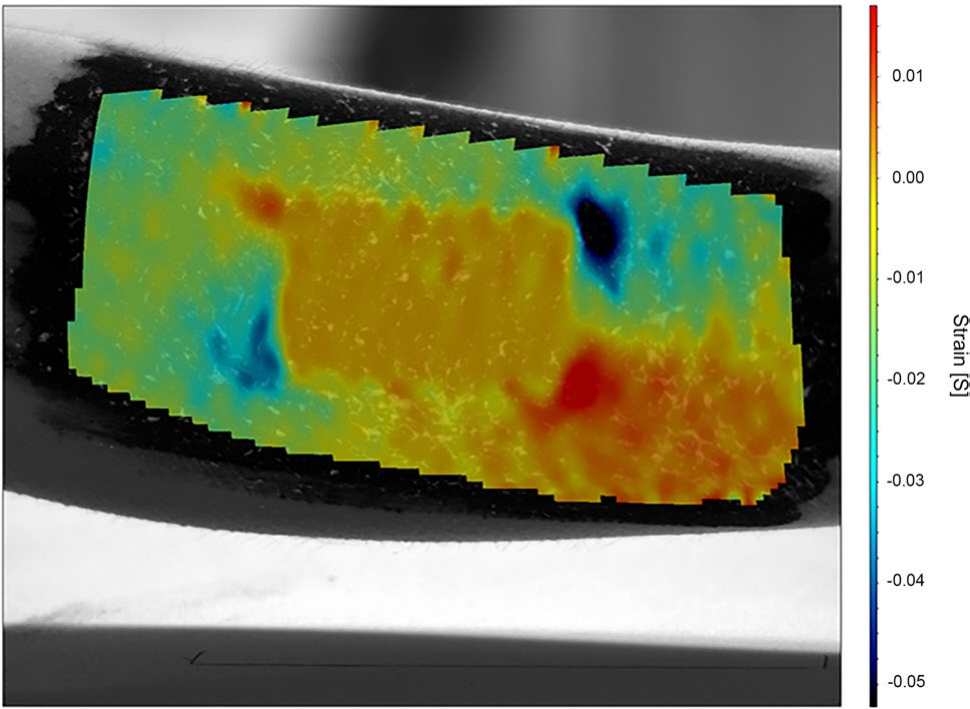
Example of a strain field for the Transpore surgical tape specimen attached to the skin. Transpore was the least deformable material in the study showing the worst-case scenario. The orange rectangle in the middle indicates the location of the sample, and the largest strains are focused mostly at the corners of this area, i.e. at the darkest red and blue areas (positive and negative strains, respectively).

The full field DIC measurements of the strain experienced by the samples and the surrounding skin visualize and quantify the local discontinuities and other sources of discomfort on the skin. The results shed some light on the requirements on e.g. the flexibility of the samples. During the measurement, the arm was rotated (i.e. twisted) to the clockwise and counterclockwise extremes, and the strains on the skin and on the device were measured during the movement. The strain and displacement results oscillate with the turning of the hand in each direction with the neutral or reference point at the center. The amplitudes of the oscillations or maximum strains during the movement of the arm were obtained for each of the materials and compared to maximum strains obtained for plain skin without any devices attached to the corresponding area. The strains observed for both the samples and the plain skin do not appear to differ much as the strains of plain skin are very small to start with (0.004–0.008). The specimens deform slightly less (0.003–0.005), the supercapacitors being at the middle of the range, Transpore and Mefix at the lower end and plain Opsite Flexifix without additional laminates and prints straining the most. The range of the observed motion was approximately a 120° turn in total. The maximum turn was limited by the field of view of the cameras. The total strains are small in all samples as well as on the plain skin. Additionally, even small local strain could cause irritation, pinching, or other discomfort. Thus, it is important that there are no significant localizations of strain either, especially around the corners of the patch on the skin. In this study these local deformations were very moderate as well, as can best be seen in full field strain images, such as the example illustrated in Fig. 7 for vertical strain (results in other directions were similar).

The DIC measurements were performed to quantify the strains the devices might experience. The results are shown here as a demonstration of the possibilities of the technique in a small case study. DIC is an excellent, contactless method for observing the deformations in these types of conditions, and there are already some studies into the strains of skin during spontaneous movement^35,36,40–42^, rather than studies of the movements induced by e.g. a torque disk attached to the skin^43,44^. However, even the studies concentrating on DIC alone are often limited in scope. There is certainly need for more comprehensive studies in the future with subjects ranging in age, sex, skin type etc. and measurements covering the entirety of the body. This is invaluable for the further development of skin mounted electronics, as the properties of the skin need to be better known if we are to design more compatible devices in the future. In earlier studies, it has been observed that even close to the large joints there are lines of non-extension where hardly any deformation takes place during even the largest of movements^35,40^. Device design plays a key part in utilizing these types of inherent properties of the body for the benefit of wearable electronics, and DIC could be one of the methods for gaining better understanding of the elastic compliance of the wearable electronics and the skin.

## Conclusions

Skin-conformable printed supercapacitors on a TPU substrate were worn attached to the skin for 24 h and electrically characterized before and after being subjected to everyday life including sleeping and changing clothes. Mechanically the samples fared well, and the patches stayed reliably in place without any complications at removal either. No leakage of liquid components was observed, and the printed current collectors and electrodes experienced no notable cracking or flaking off. The elastic compliance of the devices attached to the skin was characterized using Digital Image Correlation. DIC measurements demonstrated that the samples also strain sufficiently while attached on the skin.

Electrically, the samples survived the durability testing well and the observed aging was quite similar to the references; in some cases, as with capacitance, the wear samples even aged slightly better than the references. The results obtained from the samples after being worn on the skin had more scatter than the results obtained from the reference samples. However, the differences are small enough to be negligible for the operation of the devices. The ESR did not increase systematically even though the samples faced harsh conditions and large deformations, and the leakage current stayed within a similar range compared to those of the reference specimens, despite the slight temperature increase.

The printed supercapacitors perform well when directly attached to the skin, and their properties do not degrade when exposed to stressors arising from everyday life. The samples function reliably through the 24 h test period and their properties do not degrade during wear. The printed epidermal supercapacitor offers a novel combination of user-safe materials within a conformable packaging that is demonstrably robust enough to withstand significant mechanical stress. Device design and placement play critical roles in further applications but in light of this work and others, extreme stretchability is not a necessary prerequisite for comfortability or durability in many applications.

### Ethical statement

The study was approved by the Ethics Committee of the Tampere Region. All research involving human participants was performed in accordance with the requirements of the institutional and/or national research committee and with the Declaration of Helsinki. Only the authors of the study participated in the experiments and no external people were involved; informed consent was obtained from all the authors.

## Supplementary information

Supplementary Information.
