# Supplementary material for: Skin-conformable printed supercapacitors and their performance in wear
Source: Sci Rep. 2020 Sep 16;10:15194. doi: 10.1038/s41598-020-72244-8 (PMC7495439; doi:10.1038/s41598-020-72244-8)

Supplementary Information

Skin-conformable printed supercapacitors and their performance in wear

Anna Railanmaa, Ayat Soltani, Suvi Lehtimäki, Nazanin Pournoori, Jari Keskinen, Mikko Hokka, Donald Lupo

Figure S1. Schematic of the manufacturing sequence of the skin-conformable supercapacitor.

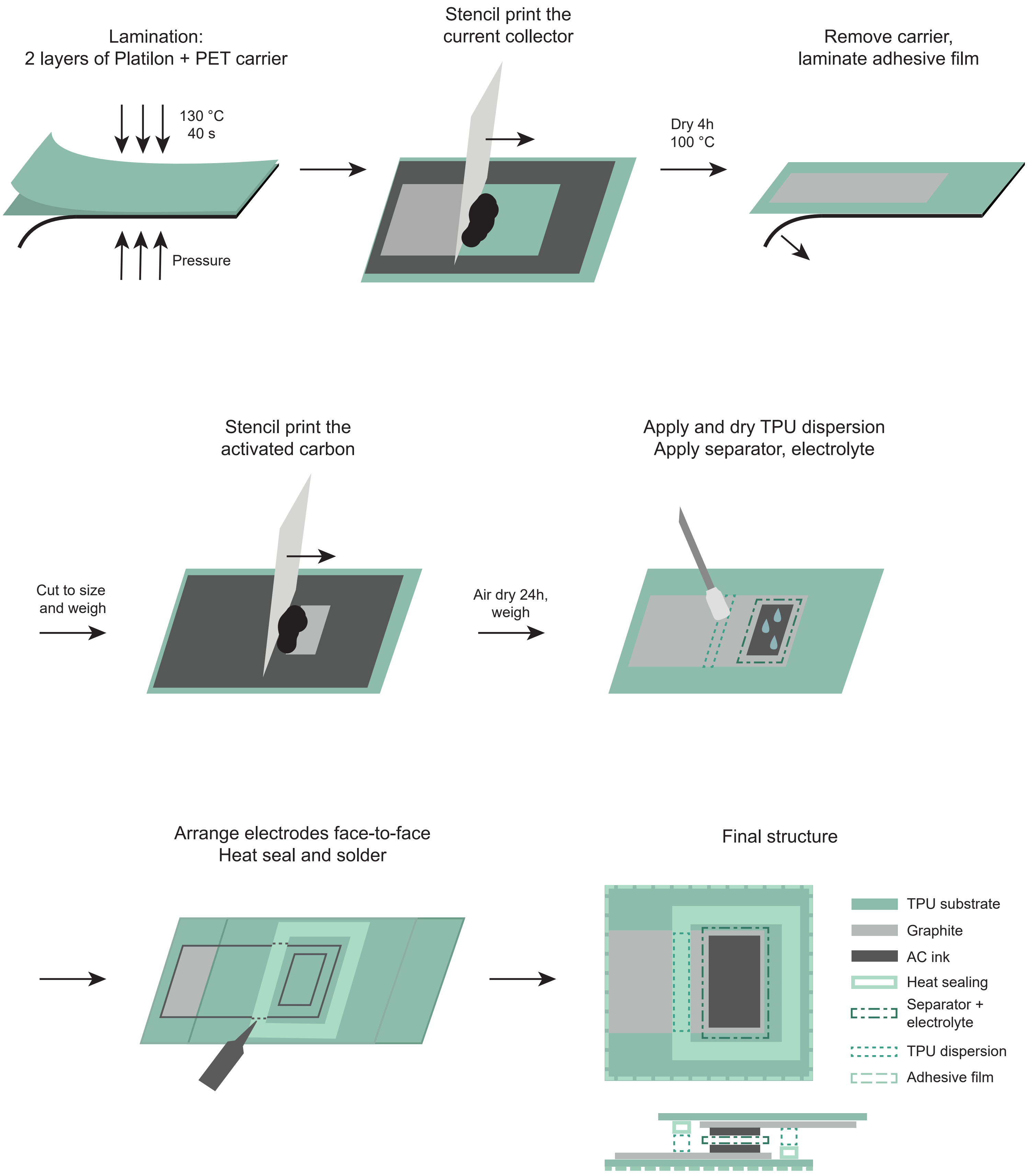

Supplement: Supplementary file 1 — Supplementary Information. [file 41598_2020_72244_MOESM1_ESM.pdf]
